# Flexural Response and Failure Analysis of Solid and Hollow Core Concrete Beams with Additional Opening at Different Locations

**DOI:** 10.3390/ma14237203

**Published:** 2021-11-25

**Authors:** Ibrahim A. Sharaky, Ahmed S. Elamary, Yasir M. Alharthi

**Affiliations:** Civil Engineering Department, College of Engineering, Taif University, Taif 21944, Saudi Arabia; a.elamary@tu.edu.sa (A.S.E.); y.harthi@tu.edu.sa (Y.M.A.)

**Keywords:** concrete beam, hollow section, flexural strength, finite element model, failure mode

## Abstract

It is essential to make openings in structural concrete elements to accommodate mechanical and electrical needs. To study the effect of these openings on the performance of reinforced concrete (RC) elements, a numerical investigation was performed and validated using previous experimental work. The effect of the position and dimension of the opening and the beam length on the response of the beams, loads capacities, and failure modes was studied. The simulated RC beams showed different responses, loads capacities, and failure modes depending on the position and dimension of the opening. The transversal near support opening (TNSH) and longitudinal holes (LH) showed lower effects on the load capacities of the beams than the transversal near center opening (TNCH). The supreme reduction percentages of the load capacity (*µ_u_*%) for beams with TNCH and TNSH were 37.21% and 30.34%, respectively (opening size = 150 × 150 mm^2^). In addition, the maximum *µ_u_*% for beam with LH was 17.82% (opening size = 25% of the beam size). The TNSH with a width of less than 18.18% of the beam shear span (550 mm) had trivial effects on the beam’s load capacities (the maximum *µ_u_*% = 1.26%). Although the beams with combined LH and TNCH or LH and TNSH showed different failure modes, they experienced nearly the same load reductions. Moreover, the length of the beam (solid or hollow) had a great effect on its failure mode and load capacity. Finally, equations were proposed and validated to calculate the yield load and post-cracking deflection for the concrete beams with a longitudinal opening.

## 1. Introduction

Hollow core concrete (HCC) beams are advantageous when compared to solid concrete beams due to the weight reduction that particularly affects the transportation cost. HCC beams can also reduce the cost of the handling and erection of precast beams. The required materials for HCC beams are significantly less than those needed for conventional beams, and they have the accessibility provision for passing electromechanical works. These advantages have motivated researchers to study the structural behavior of HCC beams under different loading conditions. Numerous studies have been conducted to analyze the behavior of hollow as well as solid beams that have the same reinforcement and cross-section under pure torsion only. When the beams are subjected to pure torsion, the obtained results verify that hollow and solid beams of the same materials and dimensions practically failed at the same loads, and the internal concrete core had minimum effect on their response [1,2,3].

However, relatively few studies have compared the failure loads as well as the behavior of the reinforced concrete (RC) beams with hollow cross-sections subjected to combined bending and torsion [1,4]. The inner concrete core has experienced no contribution to the torsional capacity of tested RC elements. In contrast, the experimental study conducted on hollow and solid RC beams in [2] showed a small effect of the hollow core on beams’ torsional capacities. The inside concrete core did not experience any part of the torsional capacity of the members. Fouad et al. [4] conducted tests on RC beams conducted with normal concrete (NC) and high-strength concrete (HSC). The results verified that the hollow core affects the cracking capacity; however, the beams with hollow core (HC) attained the same torsional resistance of RC beams with solid sections. In addition, similar initial responses were noticed for RC beams with or without an opening. For beams with HC, the cracking torque and the ultimate strengths were about 52–69% and 95–98%, respectively, compared to corresponding beams without HC [4]. Torsion tests were also performed on a set of 16 HC beams conducted with HSC [5]. The results showed that there were four different types of failures for HSC beams under torsion, which were directly linked to the reinforcement ratio. The failure types were brittle failure due to insufficient reinforcement, ductile failure, brittle failure due to corner break off, and brittle failure due to insufficient concrete strength. The results also showed some ductile behavior in a close interval of the torsional reinforcement ratios [5].

Bhatt and Ebireri [6] experimentally investigated twelve hollow and solid beams that were subjected to combined bending and torsion. The solid beams in this research were hollow-designed with the assumption that the torsion would be resisted by the external 50-mm thickness. The results showed that both beams (solid and hollow) subjected to pure torsion failed below the design loads (approximately 10%), whereas those under dominant bending failed above the design loads. Moreover, there was no general agreement on the effect of the combination of the shear, bending, and torsion loads on the behavior and ultimate load of the beams. Alnuaimi et al. [7] compared seven solid RC beams with corresponding hollow ones subjected to a combined load of bending, torsion, and shear. The results revealed that the concrete core was significantly affecting the behavior and strength of the beams when beams were subjected to the exerted combined loads (bending, torsion, and shear). The core influence partially depended on the torsion/bending moment ratio and the ratio of shear stresses (torsion/shear force). The cracks in the hollow RC beams appeared at much lower loads than for solid beams. It was also found that the solid beams were preferable when the bending load was dominating, whereas the hollow beams were preferable when torsional load was dominating. Interestingly, a three-dimensional analytical model was developed by [3] that could analyze rectangular sections subjected to a combined load of biaxial bending, biaxial shear, axial loads, and torsion. In this model, the interaction between shear and torsion as well as the concrete spalling could be considered. It idealized rectangular cross-section shapes, as formed by four transversely reinforced walls of varying thicknesses and angles, resisting the shear and torsion of the principal compressive strains. The model showed good agreement with the experimental behavior and ultimate load results. Another study was performed by [8] on the flexural response of hollow HSC beams. The cross-section for all beams was 150 × 150 mm^2^ with varying square hollow core sections (side: 60, 80, and 100 mm). The results indicated that the ductility of the hollow beams with size reductions of 16% and 28.4% were approximately 15 to 24% higher than that of solid beam.

The deflection of simply supported, rectangular RC beams with a longitudinal circular opening was also studied [9]. Seventy-six RC hollow beams along with ten RC solid beams were experimentally tested until failure. Crack patterns and deflection corresponding to the applied load were intensively observed. The results revealed that the RC beams that had a longitudinal circular opening failed by shear mode when tested with a shear span to effective depth (L_sh_/d) ratio of 1.37, by flexure–shear mode when tested with (L_sh_/d) ratio of 1.6–2.56, and by flexure mode when tested with (L_sh_/d) ratio of 2.69. The ACI equation that predicts the deflections of solid beams was modified to adapt the occupied area by the opening. For a single opening of 25, 40, and 50 mm, a theoretical model was developed to predict the first cracking load and the ultimate load of RC beams [10]. The developed equations were experimentally validated by a solid RC beam and 12 hollow RC beams loaded symmetrically at two-point loads. The results showed that beams with a longitudinal circular opening and L_sh_/d = 2.69 failed by flexural mode. Moreover, the size of the opening was inversely related to the first cracking load and the ultimate load.

The effect of the opening in hollow RC beams was also studied numerically by [11]. Five models were analyzed with a length and cross-section of 6000 mm and 600 × 600 mm^2^, respectively. The beams were solid or hollow and had three sides with varying opening sizes (100, 200, and 300 mm) repeated along the beam and tested in bending. The results showed a reduction in the ultimate loads for the HSC and ultra-high-performance concrete (UHPC) by 24% and 42%, respectively, in comparison to that for the solid beams. In addition, the side opening had a negative influence on the flexural capacity. Beams with a side opening of 100 mm responded as beams with the same hollow beam, whereas the beams with a side opening of 200 mm and 300 mm showed a low response. In addition, seven dapped end beam specimens were experimentally tested by Fouad et al. [12] to investigate the effect of the opening on the strength characteristics of the RC beams. All specimens had different numbers of openings (50 mm diameter) at different locations. In comparison with the solid beam, the results showed that the ultimate load for beams with one opening was lower than that of the solid beam by 5–24%, whereas the deflection was higher than that of solid beam by 10–32%. Doubling the number of openings reduced the ultimate load by 4.7–10% and increased the deflection by 5–12%.

Unfortunately, due to lack of research on this topic, an accepted, generalized model has not yet been developed for designing hollow beams subjected to a combined load of shear, bending, and torsion. It is extremely difficult and highly expensive to perform numerous experiments for hollow core RC beams. A much more efficient approach is to develop a refined finite element (FE) model to simulate RC beams with hollow and solid sections that can be applied over a wide range of parameters. The authors have previously performed an experimental investigation [13] wherein hollow and solid beam specimens were experimentally tested to measure and observe their behaviors under bending and shear. In this study, the behaviors of the RC beams with solid and hollow sections and with an opening near the beam center or near beam supports were numerically studied under bending and shear. The results obtained experimentally were compared with those obtained from the FE simulation performed using an FE software (ABAQUS, Hibbitt, Karlsson & Sorensen, Inc., (HKS), Providence, RI, USA) to be validated. Finally, the validated model was implemented to study the effect of beam length and opening (longitudinal opening (LH), TNCH, and TNSH with different sizes) on the behavior and load capacities of RC beams. Finally, theoretical models were presented considering the area and position of the rectangular opening to predict the maximum post-cracking deflection and yield load of RC hollow beams. The analytical and numerical results were also compared.

## 2. Experimental Program

**Figure 1:**
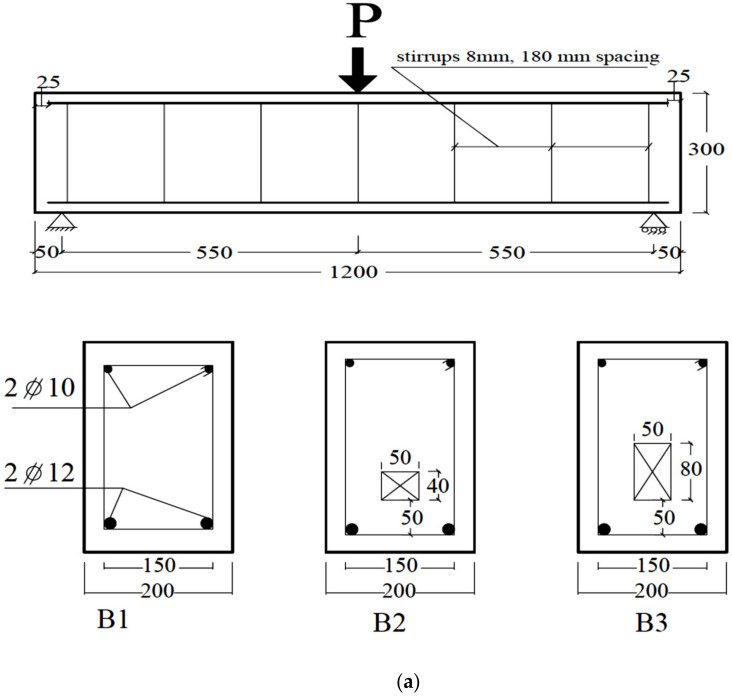

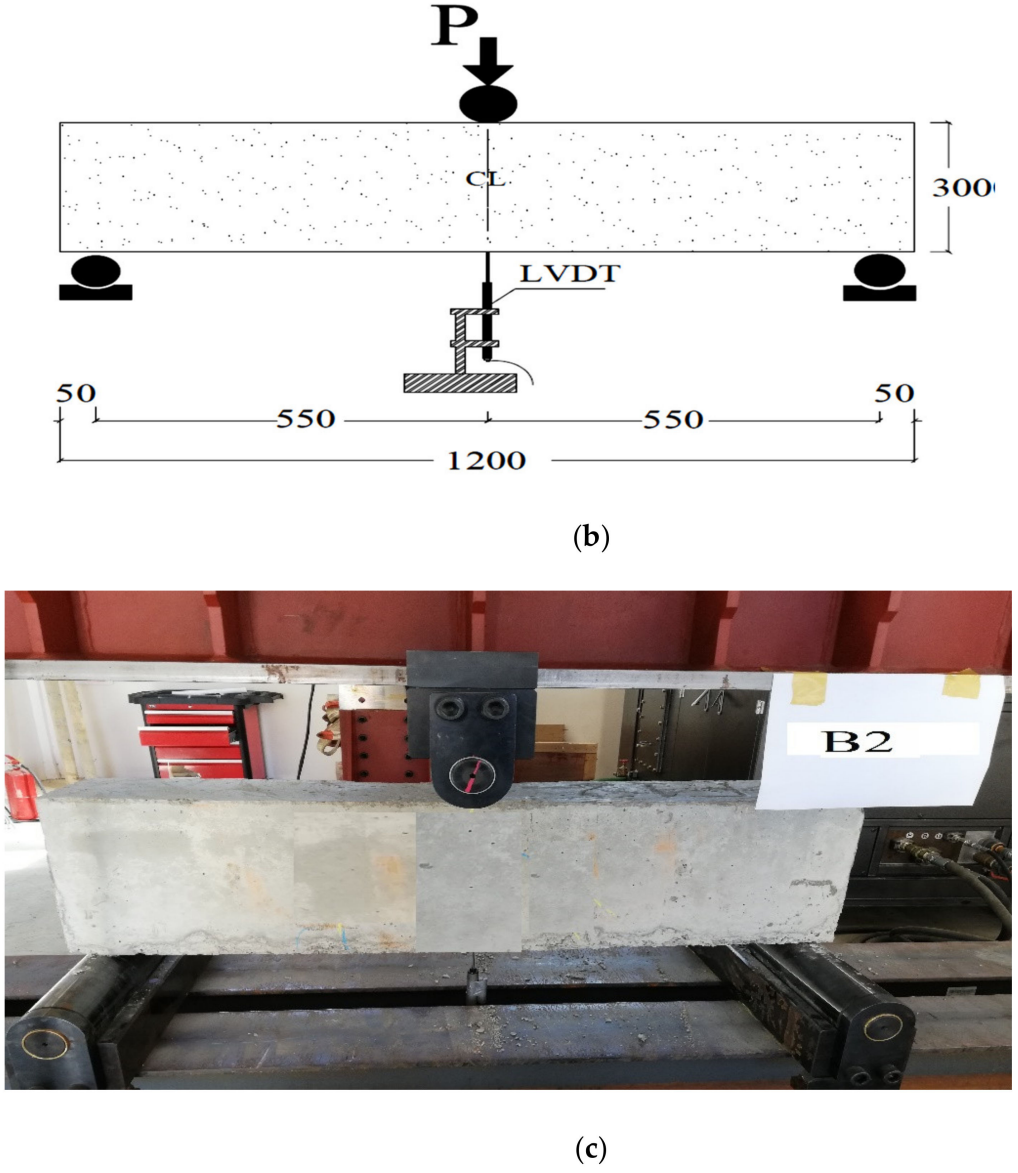
Specimens details and set up (**a**) dimensions and reinforcement, (**b**) test setup and instrumentation, and (**c**) photo of set-up and instrumentation (dimensions in mm).

The experimental work was performed by the authors and reported in detail in [13]. This experimental work is summarized herein and used to validate the numerical model that was built to perform an extensive study related to solid and hollow RC beams. According to the data reported in [13], the concrete compressive and tensile strengths (*f_cu_* and *f_ct_*) were 24 MPa and 2.64 MPa (about 11% *f_cu_* ), while the concrete modulus of elasticity (*E_c_*) was equal to 22.7 GPa. The properties of the internal steel (diameter = 10 and 12 mm) and stirrups (diameter = 8 mm) are listed in Table 1. All details of specimens’ dimensions, reinforcement, test setup, and instrumentation are illustrated in Figure 1. The length, width, and height of all beams were 1200, 200, and 300 mm, respectively.

For all beams, the longitudinal tensile reinforcement consisted of two steel bars (φ = 12 mm), whereas their compression reinforcement included two bars (φ = 10 mm). The ratio of the bottom tensile reinforcement (q = As/(bw ds)) was approximately 0.004. As illustrated in Figure 1, closed, ribbed steel stirrups (φ = 8 mm @180 mm spacing) were distributed along the beam span as shear reinforcement. The first beam (B1) was made without a hollow core (control beam, CB). The second beam (B2) was formed with a hollow cross-section area with dimensions of 50 mm × 40 mm. The third beam (B3) was formed with a hollow core with dimensions of 50 mm × 80 mm. All the dimensions and details of the opening are illustrated in Table 2 and Figure 1. The RC beams had a 1100 mm clear span and were experimentally tested under the three-point bending load. A servo-controlled hydraulic jack (2000 kN capacity) was used to load the beams with a displacement-controlled loading scheme (rate = 0.6 mm/min). Moreover, a linear variable differential transformer (LDVT with 100-mm gauge length) was fixed to capture the beam’s mid-span deflection, additional information is stated in [13].

## 3. Test Results and Discussion

**Figure 2:**
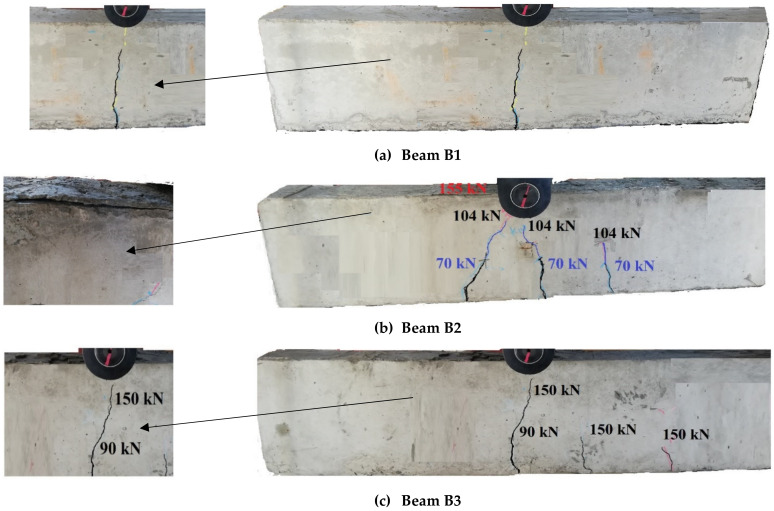
Failure modes of the tested beams.

The flexural tests were conducted on all the previously described RC beams. This section explains and discusses the obtained test results, including the loads capacities, flexural behaviors, and failure modes of the tested beams (Table 3). The symbols *P_cr_*, *P_y_*, *P_u_*, *µ_u_*%, and *w_cr_* are the cracking load, yielding load, ultimate load, reduction percentage in the load capacity ($\mu_{u}\%=\frac{P_{u,any}-P_{u,\;B1}}{P_{u,B1}}\times 100$), and the cracking width, respectively. *P_u,any_* and *P*_*u,B*1_ are the load capacity of the any beam and that of B1 (CB), respectively. Despite the differences in LH size, all the tested beams displayed similar initial failure (steel yielding, Ys). The final failure was changed to concrete crashing (CC) according to the size of LH. For all tested beams, the cracks were initiated at the beam mid-span at an approximately similar load, and they propagated vertically as the load increased. For beam B1, one crack at mid-span was created and propagated until flexural failure of the beam due to steel yielding (Ys, Figure 2).

For B2 and B3, at the beginning of the loading (approximately 39.2 and 38.8 kN, respectively), the first cracks initiated at the bottom surface of the beam (tension side). As the load raised, more flexural cracks formed near the beam mid-span. For beam B2, the previous cracks propagated towards the loading point. Subsequently, as the load increased to 70 kN, the mid-span vertical cracks became slightly wider and deeper (Figure 2b). After the load reached 104 kN, the crack length remained constant until failure whereas the crack width increased due to the bottom steel elongation as the load increased. Finally, the beam failed due to CC at *P_u_* = 155 kN, the load decreased, and the loading was stopped (Figure 2b). For B3, the mid-span crack length remained constant at a load *p* = 150 kN when more flexural and shear cracks became visible (Figure 2c). The reduction in *P_u_* of the beams with LH ranged between 3.1% and 6.3% compared to the reference beam (CB). The LH height (*h*_1_) may have changed the neutral axis position as the concrete size in the tension zone decreased. Consequently, as *h*_1_ increased, the neutral axis moved towards the tension side and increased the bottom steel stresses. As the bottom steel stresses increased, its elongation increased, and the mid-span crack width increased. The failure loads for beams B1, B2, and B3, as well as the reduction percentages relative to the CB, are listed in Table 3.

## 4. Numerical Modelling

### 4.1. Finite Element Modelling of RC Beam

**Figure 3:**
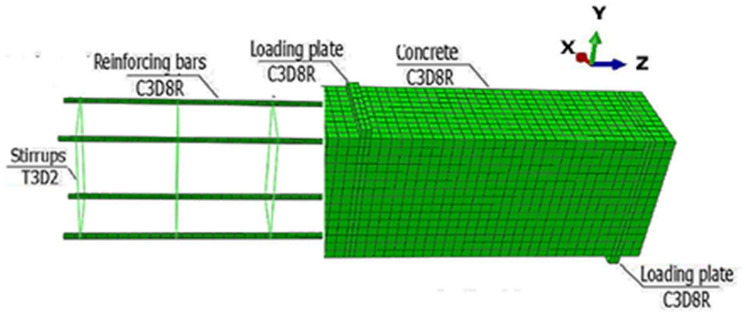
Beam modelling, mesh, and boundary conditions.

**Figure 4:**
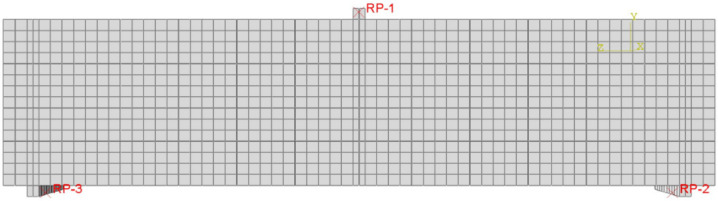
Boundary condition for the simulated beams.

A three-dimensional (3D) finite element (FE) model was built using ABAQUS [14] to simulate the previously tested beams. An eight-node linear brick element (C3D8R) with decreasing integration and hourglass control features was utilized to represent the concrete and steel reinforcement. The two-node linear 3-D truss element (T3D2) was utilized to mimic the steel stirrups [15]. Based on the sensitivities study performed with various mesh sizes (15, 20, 30, and 40 mm), a mesh size of 20 mm was chosen for all beams. The number of nodes and elements was determined by the length of the beam and the size of the aperture. The mesh was finer at the loading positions (supports and the loading point). Moreover, due to its small cross-sections, the mesh of the internal reinforcement through its cross-section was also finer than the beam mesh. The mesh was created following the recommendations proposed in [15,16,17]. The details and mesh of the model are shown in Figure 3, while the boundary condition is illustrated in Figure 4.

The tie constraints (rigid body) defined by tie nodes were used to simulate the beam loading constraints. The reference point (RP-1) was used to apply the load while the two reference points (RP-2 and RP-3) were used to apply the supporting boundary conditions. RP-1 was used to apply the load using the automatic displacement control (opposite to Y direction). RP-2 was restricted in the three directions (x, y, and z) to simulate the hinge constrain of the right support while RP-3 was restricted in x and y directions only to simulate the roller constraint at the left support. The applied load of the beam was calculated as the summation of the reactions at the two reference points RP-2 and RP-3. To simulate the interaction between the concrete and the internal steel reinforcements, the tie constraint (embedded region type) was used. The steel reinforcements (main bars and stirrups) were the embedded regions, while the concrete part was the host region. The numerical and experimental *P*–*δ* curves of all beams were compared to validate the numerical model.

### 4.2. Material Modelling

The concrete was simulated using the existing concrete model (concrete damaged plasticity, CDP) provided in ABAQUS [14,15,16,17]. The capability to model the concrete and other quasi-brittle materials is offered for various structures such as solids, shells, and beams. The concept of isotropic damaged elasticity is combined with isotropic tensile and compressive plasticity to represent the inelastic behavior of concrete. This concept is successfully used for modeling plain concrete (PC) and RC with a rebar. In this model, the compressive crushing and tensile cracking of the concrete material were considered as the main two failure mechanisms [14,15,16,18]. The material parameters associated with the CDP model were the flow potential eccentricity (ξ = 0.1), stress ratio (σ_bo_/σ_co_ = 1.16), Kc = 0.667, and dilatation angle (ψ = 35) [14,19,20]. The stress–strain compression relationship as well as the tensile post-cracking behavior of concrete were modelled according to [21,22]. The two failure/fracture options (DAMAGEC and GAMAGET) available in ABAQUS were selected to define the failure of concrete in compression and tension, respectively. The steel bars’ behavior in this model was defined as traditional elastic–plastic materials with strain hardening. For both compression and tension steel, the bilinear stress–strain relationship was used. At the supporting and loading points, loading steel plates (20 mm × 20 mm cross-section) were attached to the RC beam to avoid stress concentration.

### 4.3. Validation of the FE Model

**Figure 5:**
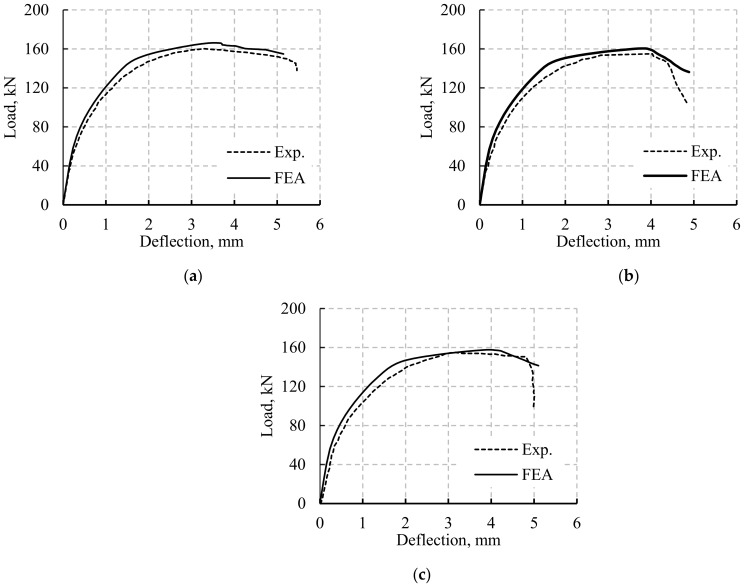
Compression between the experimental and FEA model results (verification): (**a**) Beam B1; (**b**) Beam B2; (**c**) Beam B3.

The experimental results were used to validate the numerical models with various LH configurations. The obtained FEA results were compared to the experimental ones (Exp. Results) as reported in Table 4. *P_y,Exp._*, *P_u,Exp._*, *P_y,FEA_*, and *P_u,FEA_* are the experimental yield load, experimental ultimate load, numerical yield load, and numerical ultimate load, respectively. The two terms $\frac{P_{y,Exp.}-P_{y,FEA}}{P_{y,Exp.}}\times 100$ and $\frac{P_{u,Exp.}-P_{u,FEA}}{P_{u,Exp.}}\times 100$ are the percentage difference between the experimental and numerical results at the yield and ultimate loads, respectively. Moreover, Figure 5 shows the comparison between the *P–δ* curves obtained from the experimental and FE analysis for the tested beams. A good agreement between the FEA and the experimental results were achieved in terms of stiffness and *P_u_*, as shown in Table 4 and Figure 5. The FE model appropriately reflected the non-linearity of the global behavior of the beams (Figure 5). The behavior of the RC beams was non-linear with three different stages.

**Figure 6:**
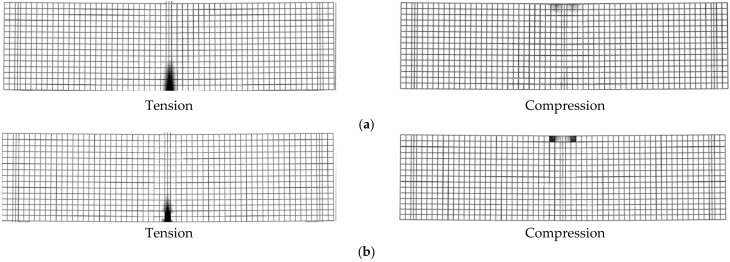
Failure modes of the simulated beams: (**a**) Beam B1; (**b**) Beams B2 and B3.

The first stage was linear and related to the elastic behavior of the materials; this was followed by a non-linear stage in which cracks appeared and propagated sequentially. The tension reinforcement of simulated beams yielded at nearly the same experimental load (maximum difference = 2.5%), whereas there were slight differences in the *P_u_* values between the FE and experimental results, as illustrated in Table 4 (ranging between 1.1% and 3.2%). The maximum difference between *P_u,exp_* and *P_u,FEA_* for CB was 3.2%. The *P_u,exp_* and *P_u,FEA_* differences were 3.0% and 1.1% for B2 and B3, respectively. The previous *P_u,exp_* and *P_u,FEA_* comparisons assured the capability of the FE model to predict the response and failure modes of the solid and LH beams. Figure 6 shows the FE fracture characteristic of the beams. The FE failure, appearance, and propagation of the cracks until the failure load of the beams followed the experimental findings (Figure 2 and Figure 6). All the beams were initially failed due to Ys. The failure of the two beams B2 and B3 was followed by concrete crushing after Ys (Figure 6b). As the opening height (*h*_1_) increased, the steel stresses amplified and the load capacity lessened. Moreover, the crack width (*w*) enlarged as *h*_1_ increased. For beams B2 and B3, after maximum load the stiffness of the beams decreased (due to cracking and steel yielding), the natural axes moved towards the top of the beams, the stress on the concrete increased, the loads of the beams decreased rapidly, and the beams failed at deflection values slightly lower than that of B1.

### 4.4. Parametric Study

**Figure 7:**
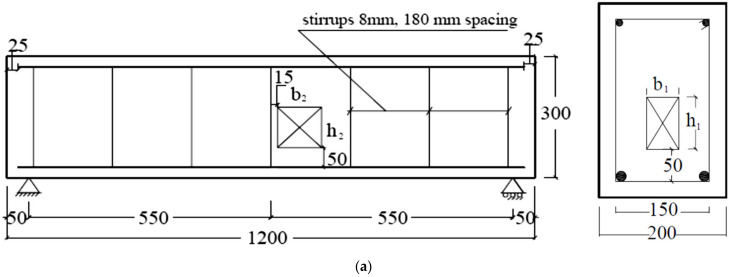

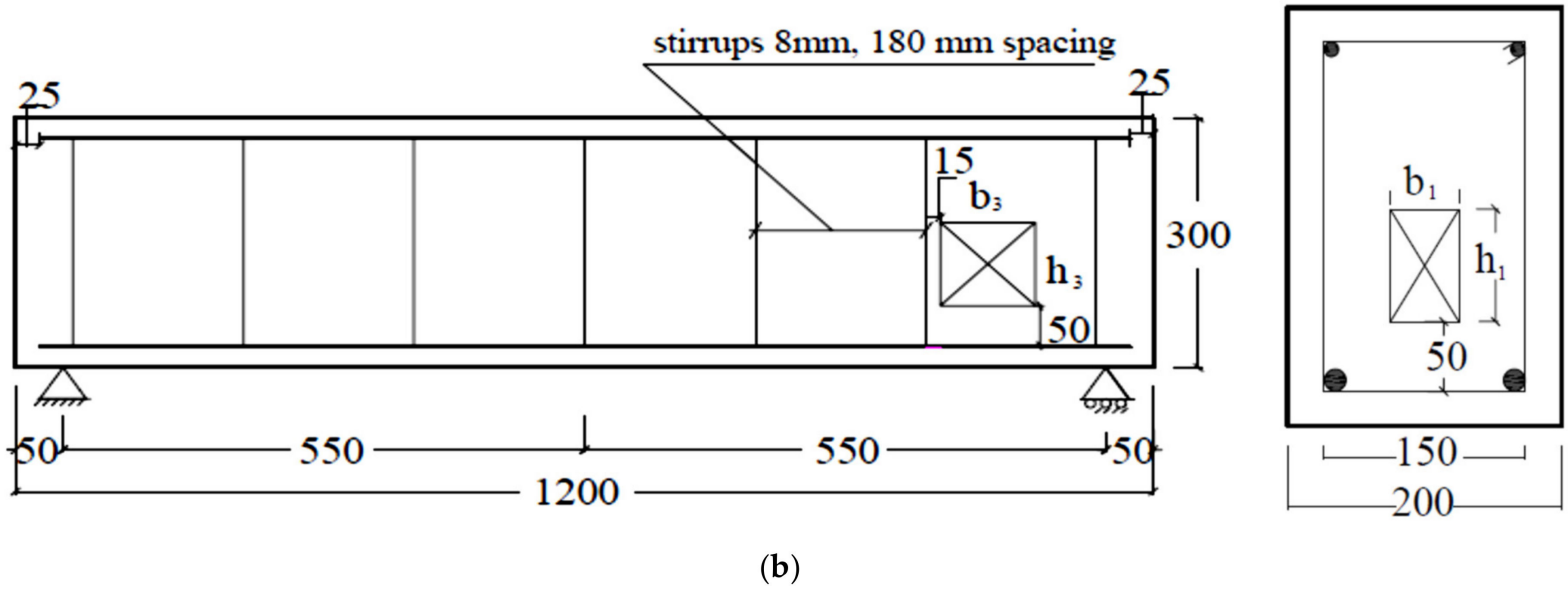
Details for the RC beams with opening in different positions: (**a**) TNCH; (**b**) TNSH (dimensions in mm).

In the present parametric study, the variables considered were the dimension and position of the opening and the beam length, while the beam internal reinforcements and cross-sectional dimensions remained constant (Figure 7). The effect of the position, opening dimension, and span length on the beam response, loads (*P_cr_*, *P_y_*, and *P_u_*), and failure modes were studied. Beam B4 had the same characteristics as B1 but with a clear span length of 2200 mm. The beam B4 was simulated to study the effect of span length on the beam’s behavior, load capacity and failure modes. Another ten groups of solid and hollow beams with an opening in different positions were also simulated. In the first group, six beams (B5-1 to B5-6) were numerically studied to discuss the effect of the LH dimensions (*h*_1_ and *b*_1_). In the second group, six beams (B6-1 to B6-6) were modeled to study the effect of the opening dimensions formed near the beam center (TNCH). In the third group, the effect of the opening’s dimensions located near the support (TNSH) were studied (B7-1 to B7-6). In group four, the combination effect of the longitudinal core and the near center opening dimensions were studied (B8-1 to B8-3). In group five, the effect of the longitudinal core and the near support opening dimensions were studied (B9-1 to B9-3). Finally, for groups 6–10, the same previous parameters presented in groups 1–5 were also studied on beams that had twice the span length of B1 (B4, L = 2200) mm. The full details of the factors studied are presented in Figure 7, while the details of beam dimensions and opening dimensions are reported in Table 5.

## 5. Results and Discussion of the Parametric Study

### 5.1. Failure Modes and Load Capacities

The numerical results of the simulated beams with solid, LH, TNCH, and TNSH are reported in Table 6. The effects of the opening’s width, depth, and position on *P_cr_*, *P_y_*, *P_u_*, and failure mode of the simulated beams are also summarized in Table 6. The terms *P_cr,FEA_*, *P_y,FEA_*, and *P_u,FEA_* are the FE cracking, FE yielding, and FE ultimate loads, respectively, while $\mu_{cr}\%=\frac{P_{cr,FEA}\left(CB\right)-P_{cr,FEA}\left(any\right)}{P_{cr,FEA}\left(CB\right)}\times 100$, $\mu_{y}\%=\frac{P_{y,FEA}\left(CB\right)-P_{y,FEA}\left(any\right)}{P_{y,FEA}\left(CB\right)}\times 100$, and $\mu_{u}\%=\frac{P_{u,FEA}\left(CB\right)-P_{u,FEA}\left(any\right)}{P_{u,FEA}\left(CB\right)}\times 100$ are reduction percentages in *P_cr,FEA_*, *P_y,FEA_*, and *P_u,FEA_* of the simulated beams, respectively. The CB refers to the beams without an opening (B1 = CB_1_ or B4 = CB_2_). In Table 6, *µ_cr_*, *µ_y_*, and *µ_u_* were calculated for beams with openings and their corresponding CB. From the table, for RC beams located in group 1 (with TNCH), as the opening height (*h*_2_) increased, the beam loads (*P_cr,FEA_*, *P_y,FEA_*, and *P_u,FEA_*) decreased. The maximum values of *µ_cr_*, *µ_y_*, and *µ_u_* were 42.41%, 23.24%, and 28.24%, respectively, for beam B5-3 (TNCH size = 100 mm × 150 mm). As the opening width increased from 100 mm to 150 mm, the beam loads also decreased. The extreme values of *µ_cr_*, *µ_y_*, and *µ_u_* were 48.19%, 32.38%, and 37.21%, respectively (B5-6 with TNSH size = 150 mm × 150 mm).

**Figure 8:**
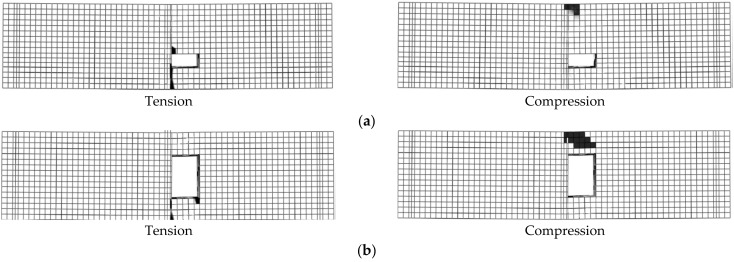

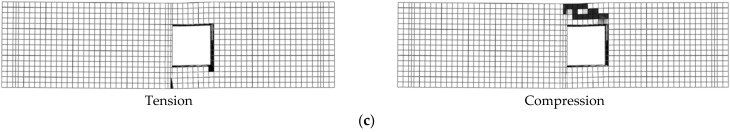
Failure modes of beams with transversal near center opening (group 1): (**a**) Beam B5-1; (**b**) Beam B5-3; (**c**) Beam B5-6.

All simulated beams located in group 1 (B5-1 to B5-6) failed due to Ys followed by CC except for beam B5-6 (failed with CC), as seen in Figure 8. The effect of opening height (*h*_2_) on the beam loads was higher than the opening width (*b*_2_) as the value of *h*_2_ affected the neutral axis position and the size of the concrete compressive part (Table 6). For simulated RC beams located in group 1 (with TNCH), as the opening height (*h*_2_) increased, the beam loads reduced. For beams having TNCH width equals 100 mm, the maximum value of *µ_cr_*, *µ_y_*, and *µ_u_* were 11.08%, 4.64%, and 1.26%, respectively (B5-3, TNCH size = 100 mm × 150 mm). As the *b*_2_ value increased from 100 mm to 150 mm, the beam loads reduced. The maximum values of *µ_cr_* and *µ_u_* for beams with *b*_2_ = 150 mm were 21.25% and 30.34%, respectively (B5-6, TNCH size = 150 mm × 150 mm).

**Figure 9:**
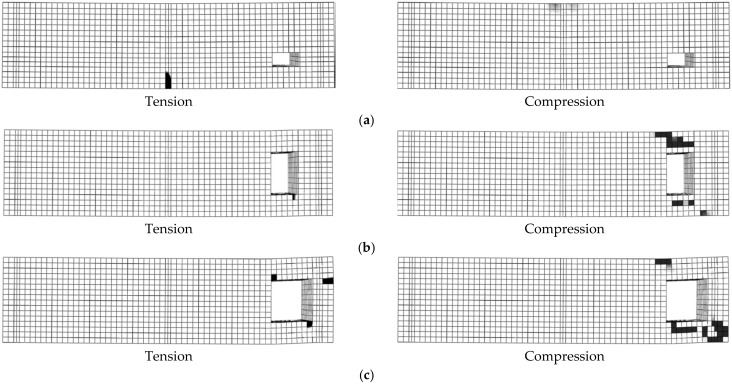
Failure modes of beams with transversal near support opening (group 2): (**a**) Beam B6-1; (**b**) Beam B6-3; (**c**) Beam B6-6.

All simulated beams located in group 2 (B6-1 to B6-6) failed due to SH except for beam B6-1 (failed with Ys), as seen in Figure 9. The effect of *b*_3_ on the beam loads was higher than *h*_3_ as the value of *b*_3_ highly affected the shear capacity of the beam. For beams with *b*_3_ = 100 mm, the maximum values of *µ_cr_*, *µ_y_*, and *µ_u_* were 11.08%, 4.64%, and 1.26%, respectively (B6-3, TNSH size = 100 mm × 150 mm). As *h*_3_ values increased from 100 mm to 150 mm, the values of *P_cr_* and *P_u_* decreased. For beams with *b*_3_ = 150 mm, the maximum values of *µ_cr_* and *µ_u_* were 21.2% and 30.34%, respectively (B6-6, TNSH size = 150 mm × 150 mm). All simulated beams with TNSH failed due to SH except for beam B6-1 (failed with Ys).

**Figure 10:**
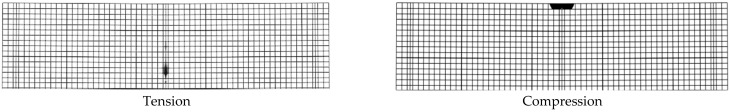
Failure modes of beams with longitudinal opening (group 3).

For simulated RC beams located in group 3 (with LH), as the opening height (*h*_1_) increased, the beam loads reduced. For beams with *b*_1_ = 80 mm, the maximum values of *µ_cr_*, *µ_y_*, and *µ_u_* were 12.77%, 11.04%, and 12.58%, respectively (B7-3, LH size = 80 mm × 150 mm). As the *h*_1_ value increased from 80 mm to 100 mm, the values of *P_cr_*, *P_y_*, and *P_u_* decreased. For beams with *b*_2_ = 100 mm, the maximum values of *µ_cr_*, *µ_y_*, and *µ_u_* were 17.83%, 14.31%, and 17.82%, respectively (B7-6, LH size = 100 mm × 150 mm). All simulated beams located in group 3 (B7-1 to B7-6) failed due to Ys followed by CC (Figure 10).

**Figure 11:**
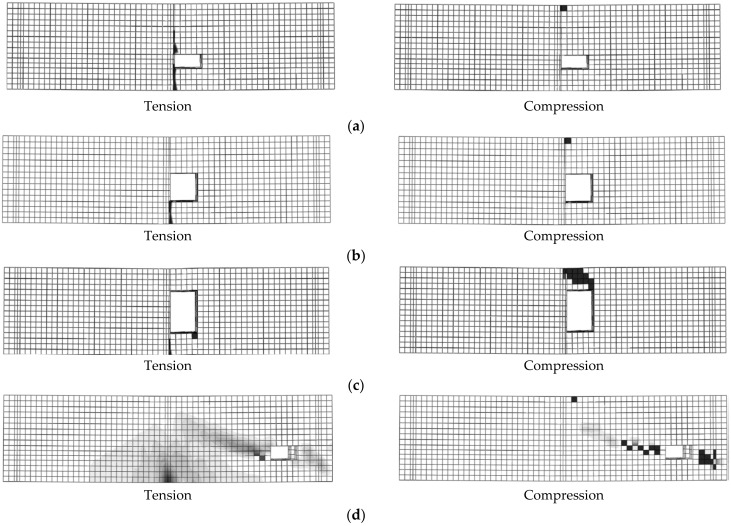
Failure modes of some beams listed in groups 4 and 5: (**a**) Beam B8-1; (**b**) Beam B8-2; (**c**) Beam B8-3; (**d**) Beam B9-1.

For simulated RC beams located in group 4 (having LH and TNCH), as the opening heights (*h*_1_ and *h*_2_) increased, the values of *P_cr_*, *P_y_*, and *P_u_* decreased (Table 6). The maximum values of *µ_cr_*, *µ_y_*, and *µ_u_* were 49.64%, 22.78%, and 27.39%, respectively (B8-3, where *b*_1_ = 80 mm, *b*_2_ = 100 mm, *h*_1_ = 150 mm, and *h*_2_ = 150 mm). All simulated beams located in group 4 (B8-1 to B8-3) failed due to Ys followed by CC (Figure 11). The value of *P_cr_* was highly affected by the opening size (Table 6). Moreover, for simulated RC beams located in group 5 (with LH, *b*_1_ = 80 mm) and TNSH (*b*_3_ = 100 mm), as the opening heights (*h*_1_ and *h*_3_) increased, the values of *P_cr_* and *P_u_* decreased (Table 6). The maximum values of *µ_cr_* and *µ_u_* were 24.10% and 27.27%, respectively (B9-3, *b*_1_ = 80 mm, *b*_3_ = 100 mm, *h*_1_ = 150 mm, and *h*_3_ = 150 mm). All simulated beams located in group 5 (B9-1 to B9-3) failed due to SH (Figure 11d).

**Figure 12:**
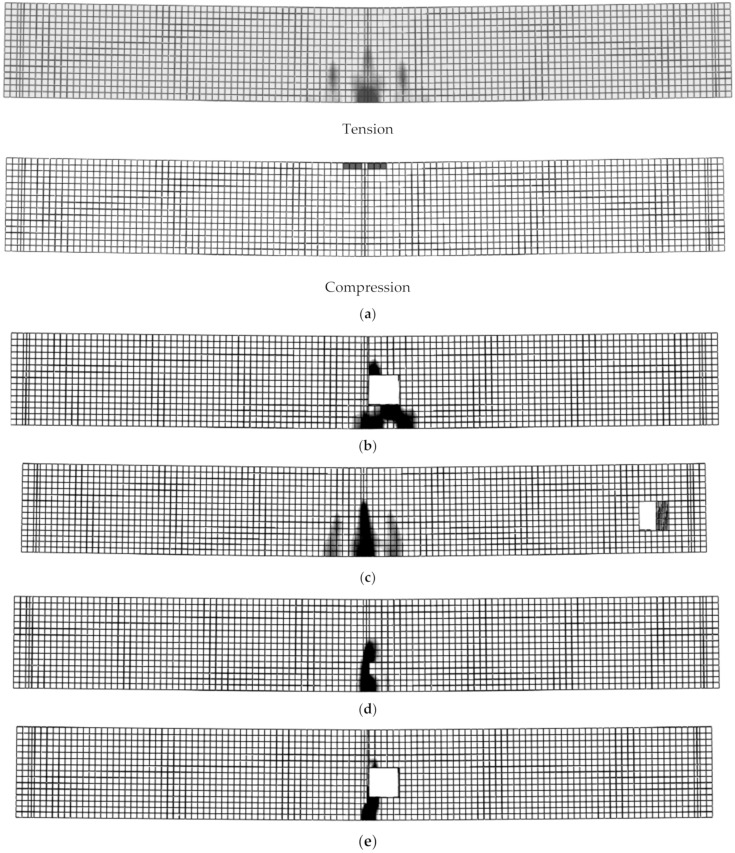

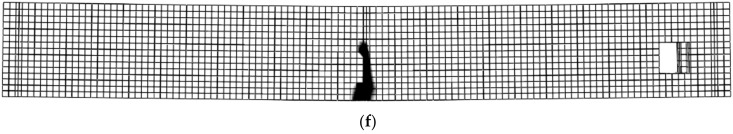
Failure modes of some beams listed in groups 10–14: (**a**) Beam B4; (**b**) B10-2 (Tension); (**c**) B11-2 (Tension); (**d**) B12-2 (Tension); (**e**) B13-2 (Tension); (**f**) B14-2 (Tension).

To study the effect of beam length on load capacity and failure modes, beams with 2200 mm span length and with openings in different positions were also simulated. For beams with a span length of 2200 mm, the failure of B4 and those with TNCH was similar to the corresponding beams with a span length of 1100 mm (Ys + CC) (Figure 8 and Figure 12a,b). Moreover, as beam span increased, the failure loads of all the beams decreased. Consequently, the experienced cracking load, yielding load, and ultimate load of B4 (span length = 2200 mm) were about 88.2%, 54.0%, and 58.9%, respectively, compared to those of B1 (span length = 1100 mm). The load capacities of the beams in group 6 decreased by 15.2%, 17.1%, and 20.2% for the opening heights 50 mm, 100 mm, and 150 mm, respectively, compared to B4. As a result, the TNSH had slight effects on the load capacities of beams with solid cross-sections having a 2200 mm span length, while their failure modes were Ys + CC (Figure 12c). The LH decreased the load capacities of beams with a long span length compared to the corresponding beams with short span lengths, while the mode of failure was similar.

The beams that had long span lengths and LH + TNCH showed the same behavior as the corresponding beams with short spans but with high reduction in the load capacities (Figure 12e). In contrast, beams with longer lengths and LH + TNSH failed due to Ys + CC (Figure 12f) and showed lower reduction in the load capacities compared to those having short lengths as they had failed due to SH. The reduction in the load capacities for beams with long span lengths were 13.3%, 15.7%, and 16.9%, respectively, compared to B4 when the opening heights were 50 mm, 100 mm, and 150 mm and their failure modes were Ys + CC.

### 5.2. The Load–Deflection Curves

**Figure 13:**
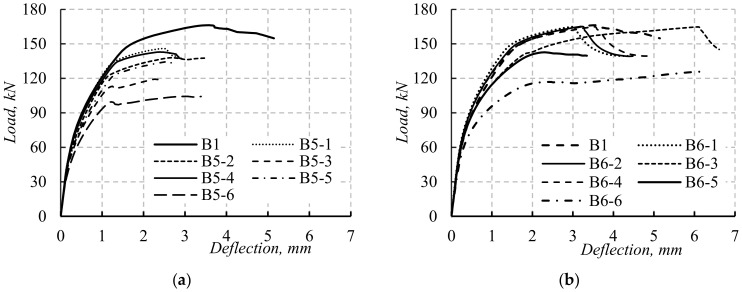

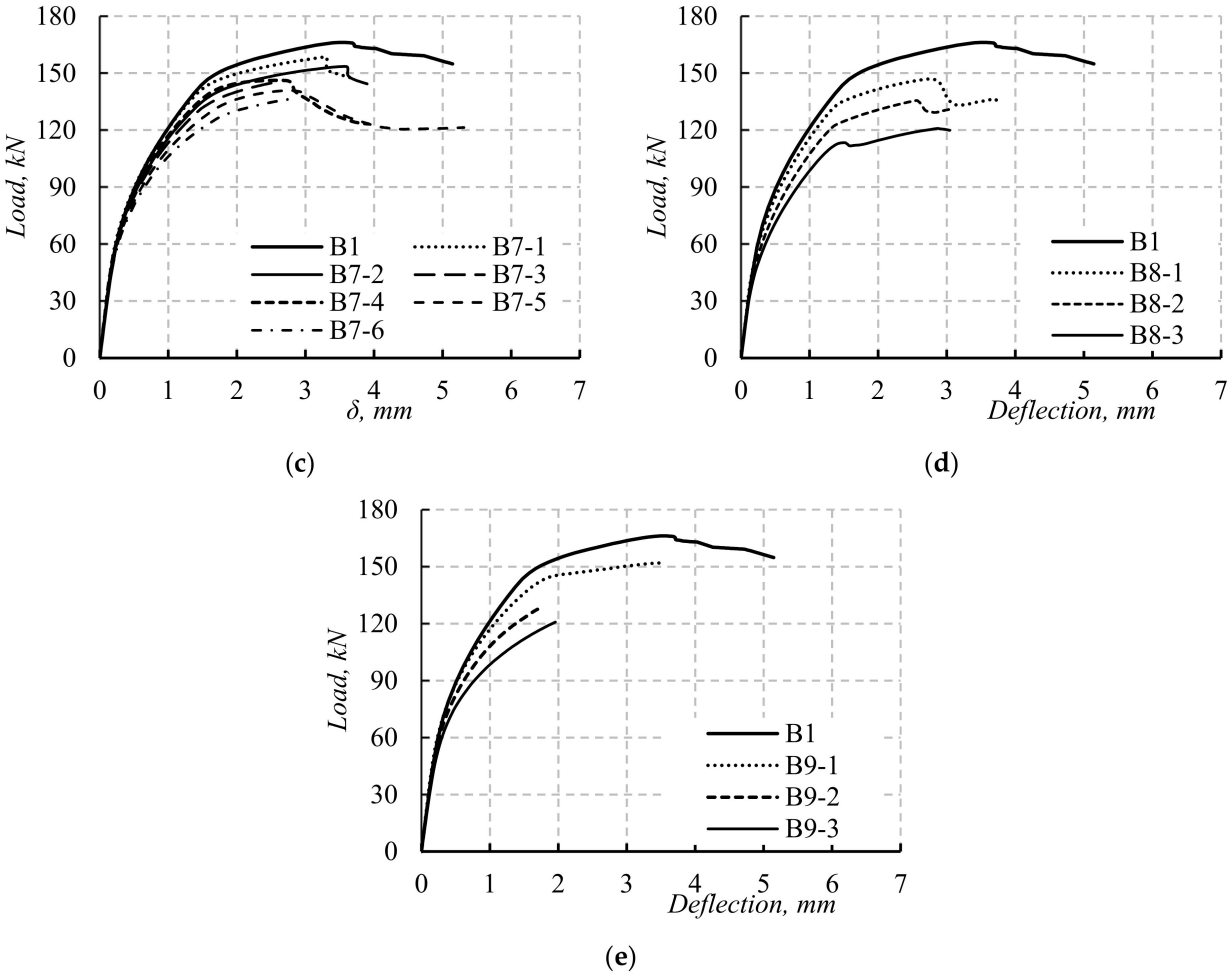
The *P*–*δ* curves of the simulated beams with L = 1100 mm: (**a**) beams with TNCH; (**b**) beams with TNSH; (**c**) beams with LH; (**d**) beams with LH and TNCH; (**e**) beams with LH and TNCH.

The load–deflection (*P*–*δ*) curves of B5-1 to B9-3 are presented in Figure 13. The beams located from group 1 to group 4 followed the same flexural response stages (three stage, Figure 13a–d). The first stage started from zero to cracking load. During this stage, all the simulated beams experienced near similar initial stiffness. The end of the first stage was dependent on *P_cr_* values affected by the opening dimensions and positions. The second stage started from *P_cr_* to *P_y_*. The stiffness of the simulated beams decreased as the opening dimension and position changed. Increasing the opening depth or width decreased both beam stiffness and *P_y_* value (Figure 13a–d). The third stage started after *P_y_* and until *P_u_*. The stiffness of simulated beams was lower than that experienced in the previous stage.

The value of *P_u_* was also affected by the dimension and position of the opening. The response of beam B9-1 was similar to the previous beams, while the response of the two beams B9-2 and B9-3 was defined by two stages only (Figure 13e). The first stage was from zero to *P_cr_*, while the second stage was from *P_cr_* to *P_u_*. The initial stiffness of beams B9-1 to B9-3 was similar to the previous beams. The stiffness decreased during the second stage. Moreover, the value of *P_u_* for simulated beams located in group 5 decreased due to the shear failure. For all simulated beams, the opening depth and width and their locations were the main factors affecting the beam response and loads.

The values of *h*_1_, *b*_1_, *h*_2_, and *b*_2_ greatly affected beam stiffness and load capacity as they affected the neutral axis of the beams. In addition, the values of *h*_3_ and *b*_3_ affected the failure loads and modes as they changed the beam failure from Ys and CC to SH. For simulated beams in groups 2 and 5, as the depth of TNSH was small, its effect on the beam load and deflection was small until maximum load capacity was reached. After the maximum load, the effect of shear on the load and deflection of the beam became obvious as the shear cracking started to deform. As the depth or width of TNSH increased, the shear strength of the beam section decreased, then its effect on increasing the deflection of the beam increased (these results agreed with those reported in [23,24]). As the size of the opening did not affect the shear capacity of the beam, the effect of shear on the total deflection of the beams could be neglected.

**Figure 14:**
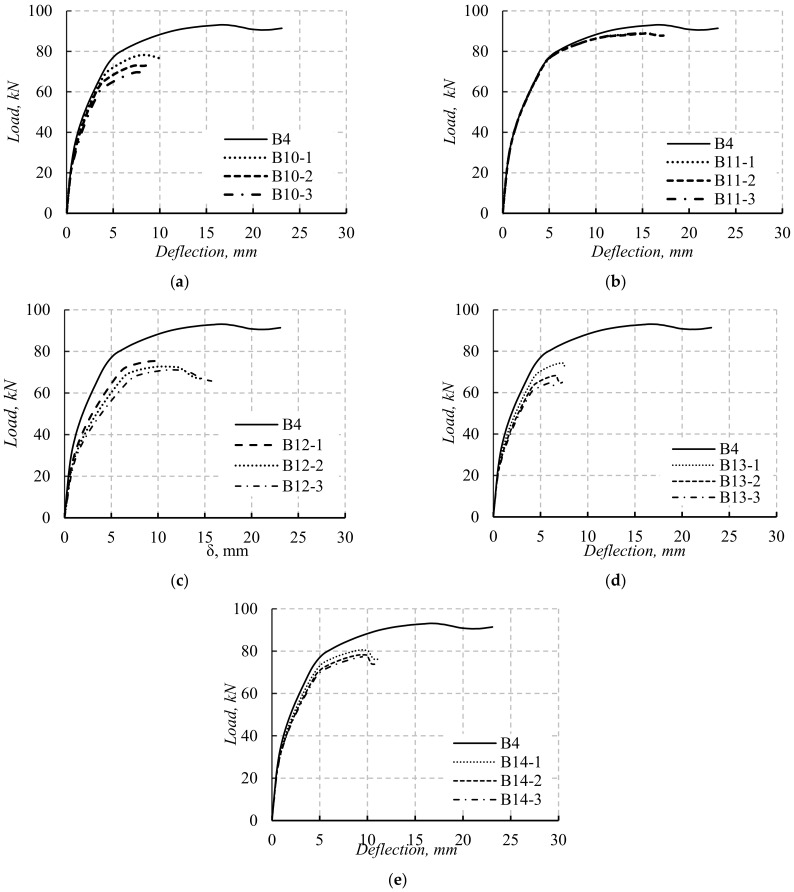
The *P*–*δ* curves of the simulated beams with L = 2200 mm: (**a**) beams with TNCH; (**b**) beams with TNSH; (**c**) beams with LH; (**d**) beams with LH and TNCH; (**e**) beams with LH and TNCH.

The effects of opening sizes on the stiffness of beams with long spans were lower than those of corresponding beams with short spans (Figure 13 and Figure 14). The opening in the beam with short spans was greatly affected by the shear deflection of these beams. In contrast, the opening had slight effects on the shear loads and deflection of the simulated beams with a long span as their failure mode was due to flexural failure only. For simulated beams with long span lengths, the effect of LH and TNCH on the loads and stiffness was higher than that of beams having TNSH as their failure was flexural failure only (Figure 14).

### 5.3. Discussion

**Figure 15:**
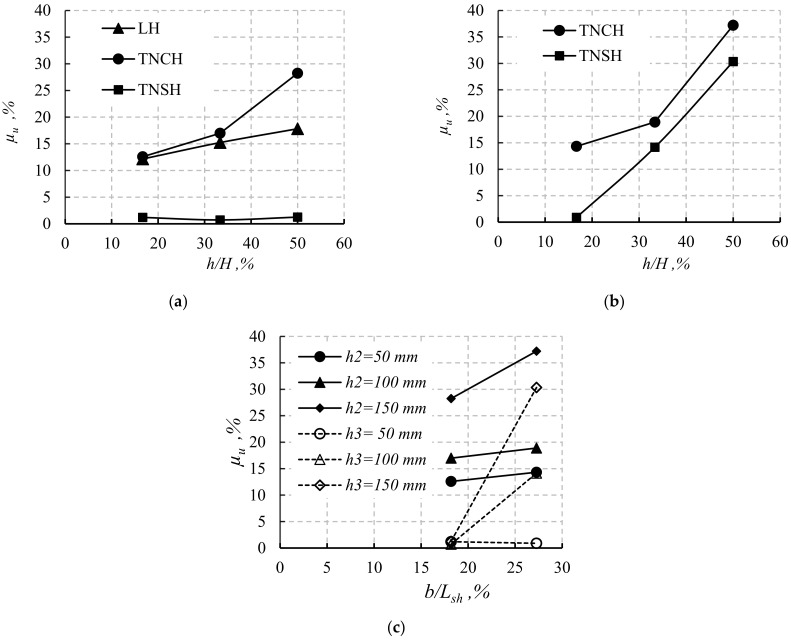
Effect of opening dimensions and position on the reduction in the ultimate load capacity for beams with *L* = 1100 mm: (**a**) effect of *h*/*H* at *b* = 100 mm; (**b**) effect of *h*/*H* at *b* = 150 mm; (**c**) effect of *b*/*Lsh*.

Figure 15 explains the effect of opening position and dimension on the value of *P_u_*. The effect of the percentage of opening height (*h*) to the total beam depth (*h*/*H*%) and opening position on the reduction percentage in *P_u_* (*µ_u_*%) of the beams with an opening is shown in Figure 15a,b. To only adopt the effect of *h*/*H*% and opening position on the *µ_u_*% and the opening, the beams with constant width (*b*) were considered (*b*_1_ = *b*_2_ = *b*_3_ = 100 mm, Figure 15a and *b*_2_ = *b*_3_ = 150 mm, Figure 15b. At *b* = 100 mm, simulated beams with TNSH experienced the lowest *µ_u_*% for all the studied values of *h*/*H*%. Moreover, the simulated beams with TNCH experienced the highest values of *µ_u_*%. These findings assured the higher effect of TNCH opening on the flexural capacity than TNSH opening. The same previous findings were observed for TNSH and TNCH openings with *b* = 150 mm (Figure 15b). The *µ_u_*% increased as *h* increased, regardless of the opening position. In contrast, the opening position greatly affected the *µ_u_*%, especially for *b* = 100 mm (Figure 15a). The opening position effects decreased as the *b* value increased to 150 mm, especially when *h*/*H*% ≥ 1/3 (Figure 15b). In addition, as *b* increased from 100 mm to 150 mm, the value of *µ_u_*% increased for the simulated beams with TNSH and TNCH (Figure 15a,b).

To discuss the effect of *b*/*L_sh_* and opening position on the *µ_u_*%, Figure 15c was drawn (*L_sh_* is the shear span length). For the same value of h, simulated beams with TNCH experienced higher *µ_u_*% than that for simulated beams with TNSH. In contrast, increasing *b*/*L_sh_* served to increase *µ_u_*% for simulated beams with TNSH with higher values than that for simulated beams with TNCH, especially for *b*/*L_sh_* = 27.27% and *h*/*H*% ≥ 1/3. Previous findings assured that the *b*/*L_sh_* affects the shear capacities of the beams rather than their flexural capacities.

**Figure 16:**
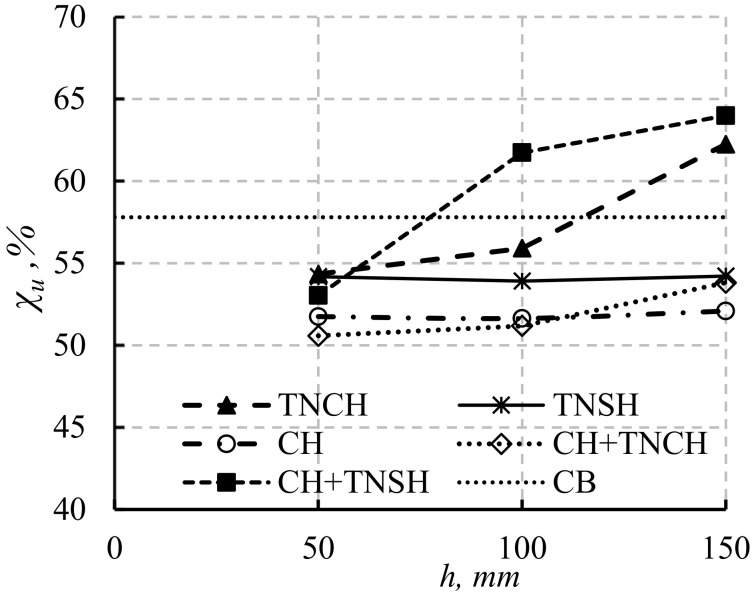
The effect of beam length on the failure loads of solid and hollow beams with openings in different positions.

The load efficiency ratios for simulated beams with span lengths of 2200 and 1100 mm ($\chi_{u}\%=\frac{\mu_{u,L}}{\mu_{u,sh}}\times 100$) and corresponding opening heights were also studied (Figure 16). *µ_u,L_* and *µ_u,sh_* are the ultimate load efficiency for simulated beams with long and short spans, respectively. The load efficiency ratio of simulated beams was slightly affected by increasing the opening height in the two cases of LH + TNSH and LH only. In contrast, the existence of LH, LH + TNCH, and TNSH decreased the load efficiency ratio compared to that of solid beams. The χ% values for simulated beams with LH + TNCH and TNSH were higher than those of solid beams when the opening heights were higher than 100 mm and 150 mm, respectively (Figure 16). These observations assured the higher effectiveness of these opening position on the loads of the short span beams compared to those of long span beams as the long span beams suffered from lower shear stresses than the short span beams.

## 6. Development of Theoretical Models

This section presents theoretical models that consider the area and position of the rectangular opening to predict the maximum post-cracking deflection and yield load of RC hollow beams.

### 6.1. Load–Deflection Relationships

**Figure 17:**
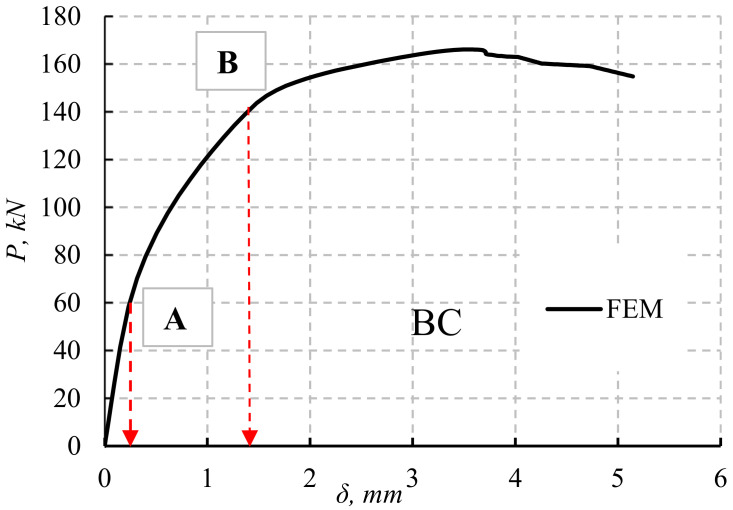
BC–load deflection curve stages.

The load versus central deflections for a typical control beam is shown in Figure 17. It can be noticed that there are three distinct types of load–deflection relations. A linear and maximum slope is observed at the beginning of the graph up to point A. This part of the graph represents the pre-cracking stage. After the initiation of cracks, the slope of the graph changes. Point B represents the load and deflection at which the yielding of steel starts to occur. Beyond point B, the graph is curvilinear.

### 6.2. Pre-Cracking Deflection Determination

**Figure 18:**
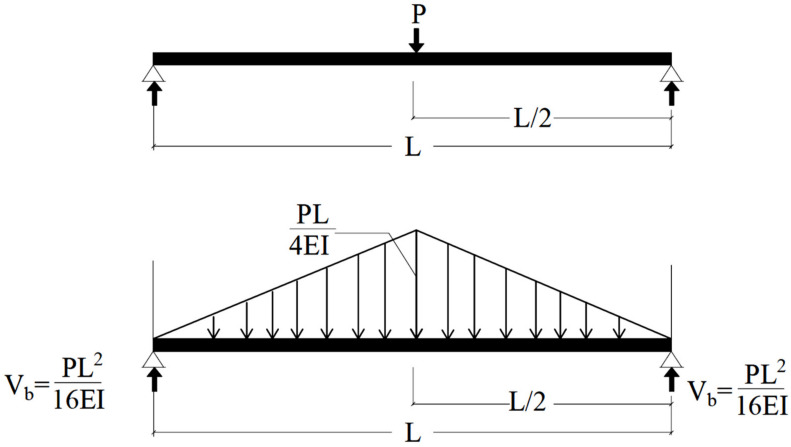
Conjugate beam loaded with M/EI diagram.

Based on the conjugate beam method, Figure 18 shows the load on the conjugate beam which equal the volume of the load diagram:

(1) $$\mathrm{Load}\;\mathrm{on}\;\mathrm{the}\;\mathrm{conjugate}\;\mathrm{beam}=\;\frac{1}{8}\frac{PL^{2}}{EI}$$

Equation (2) shows the reaction at each support of the conjugate beam:

(2) $$Elastic\;Reaction=\frac{1}{16}\frac{PL^{2}}{EI}$$

By taking the bending moment at the center of the conjugate beam (Figure 18), the deflection at the center of the beam can be obtained as follows (3):

(3) $$\delta_{pcr}=\frac{PL^{2}}{16EI}\;\frac{L}{2}-\frac{PL^{2}}{16EI}\;\frac{2}{3}\;\frac{L}{2}=\frac{PL^{3}}{48\;EI}$$

where ($\delta_{pcr}$) is the central deflection corresponding to the pre-cracking stage.

This deflection at the central point loading is evaluated using the relationship provided in Equation (4):

(4) $$\delta_{pcr}=\frac{1}{48}\frac{PL^{3}}{EI_{ucr}}$$

where *P* is the central applied load, *L* is the beam length, *E* is the modulus of elasticity, and *I_ucr_* is the moment of inertia of the uncracked section.

**Figure 19:**
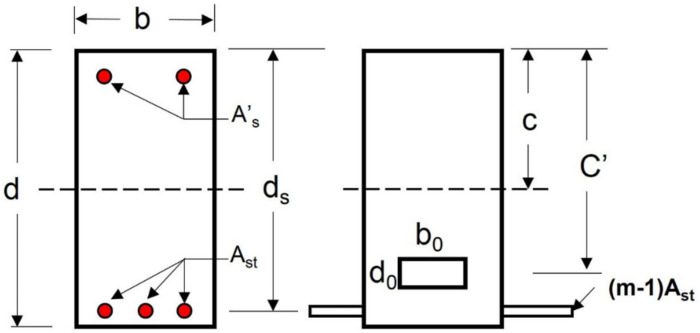
Transformed section of RC beam—before cracking.

To calculate the first cracking load, the elastic theory of simple bending and the proper ties of the transformed section (steel area transformed into equivalent concrete area) can be used. The area of the uncracked transformed section (*A_t_*) for the hollow beam section illustrated in Figure 19 is calculated using Equation (5):

(5) $$A_{t}=bd-A_{h}+\left(m-1\right)A_{st}$$

where ($A_{t}$) is the area of uncracked transformed section, $\left(A_{h}\right)$ area of opening, ($A_{st}$) area of tension steel, and (*m*) is the modular ratio. The value of (*c*), the depth of the neutral axis, is obtained by taking moment of the areas about the top edge, as in Equation (6):

(6) $$A_{t}c=bd\frac{d}{2}-A_{h}c^{\prime }+\left[\left(m-1\right)A_{st}d_{s}\right]$$

Equation (6) can calculate the minimum value of (*c*) where the value of (*c*) is the height of the compression zone and (c′) is the distance of the center of opening from the top fiber of concrete. The following Equation (7) can be used to calculate the moment of inertia of the un-cracked transformed section ($I_{ucr}$) about the neutral axis:

(7) $$I_{ucr}=\frac{bd^{3}}{12}+bd{\left(C-\frac{d}{2}\right)}^{2}+\left(m-1\right)A_{st}{\left(d_{s}-c\right)}^{2}-\left[\frac{b_{o}d_{o}^{3}}{12}-A_{h}{\left(C-C^{\prime }\right)}^{2}\right]$$

Substituting Equation (7) into Equation (4), the central deflection at the pre-cracking stage of RC hollow beams can be estimated.

### 6.3. Post-Cracking Deflection Determination

As the applied load increased, the cracks propagated and widened along the section periphery. Increasing the depth and width of the cracks meant that they were becoming closer to the neutral axis in the latter case. Between A and B (Figure 17), the depth and width of the cracks were directly proportional with the moment diagram. As a result, the actual effective moment of inertia (*I_eff_*) calculated for the cross-section would be between the uncracked value and the fully cracked value.

Branson [25,26] proposed an effective constant moment of inertia that is empirically derived to calculate deflection. This empirical formula is recommended to be used by both the American Concrete Institute (ACI) building code ACI 318- 11 [27] and the Canadian Concrete Design Standard CSA A23.3-04 [28]. Based on the cracked transformed section analysis, the empirical relationship is proposed to represent a gradual transition from the uncracked transformed moment of inertia (*I_ucr_*) to a fully cracked moment of inertia (*I_cr_*). Using this equation, the effect of reinforcement and cracks on the stiffness of a member is considered when the applied load increases. It should be mentioned that this equation cannot be used for heavily reinforced sections. In this case, (*I_ucr_*) is replaced by the gross moment of inertia (*I_g_*).

**Figure 20:**
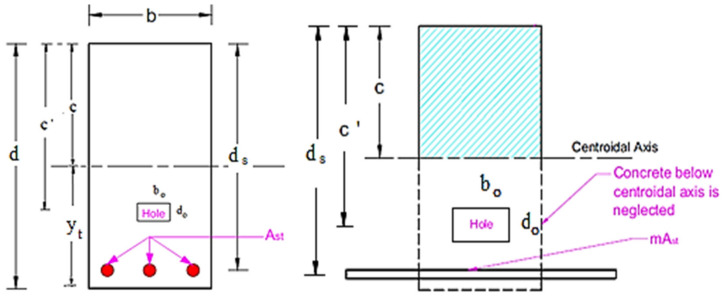
RC beam—transformed section after cracking.

For the beam section, the ratio of the uncracked to cracked transformed moment of inertia (*I_ucr_*/*I_cr_*) and the applied level of loading relative to the cracking load are the main tension-stiffening components that are affecting this method [29]. As well as this, Bischoff [30] stated that the tension-stiffening component with this method is highly dependent on the applied level of loading relative to the cracking load. The formula for the effective moment of inertia proposed by Branson and Bischoff was examined by Ilker [31]. Ilker [31] revealed that the method proposed by Bischoff provides a slightly better correlation with the actual in-plane bending deformations of heavily RC beams than the method proposed to replace the (*I_ucr_*) by (*I_g_*). In Figure 20, the depth of the neutral axis (c) can be calculated using Equations (8) and (9).

In case c′−do2 ≥c

(8) $$\frac{bC^{2}}{2}=mA_{st}\left(d_{s}-c\right)$$

In case c′−do2<c

(9) bC22−Ah″2c−c′−do2=mAstds−c

where Ah″=bo c−c′−do2.

Similarly, Equations (10) and (11) can be used to calculate the cracked moment of inertia of the RC hollow transformed section about the horizontal centroidal axis.

In case c′−do2 ≥c

(10) $$I_{cr}=\frac{bC^{3}}{3}+mA_{st}{\left(d_{s}-C\right)}^{2}$$

In case c′−do2<c

(11) Icr=bC33+mAstds−c2−bodo″ 312+Ah″0.5c−c′−do22

where do″=c−c′−do2.

From Figure 20, the location of the horizontal centroidal axis from the bottom edge can be determined using Equation (12).

By taking moments of area about the bottom edge

(12) $$y_{t}=\left[\frac{\frac{bd^{2}}{2}-\left[A_{h}\left(d-C^{\prime }\right)\right]}{\left(bd-A_{h}\right)}\right]$$

Furthermore, the moment of inertia of this section is calculated using Equation (13):

(13) $$I_{gc}=\frac{bd^{3}}{12}+bd{\left(\frac{d}{2}-y_{t}\right)}^{2}-\left[\frac{b_{o}d_{o}^{3}}{12}+A_{h}{\left(y_{t}-\left(d-c^{\prime }\right)\right)}^{2}\right]$$

Moreover, the cracking–bending moment $M_{cr}$ is provided by Equation (14):

(14) $$M_{cr}=\frac{f_{cr}\;I_{gc}}{y_{t}}$$

where *f_cr_* is the modulus of rupture value of concrete.

Based on that provided for a solid section by ACI 318-11 [27], the effective moment of inertia of the hollow beam section can be calculated using Equation (15):

(15) $$I_{eff}={\left[\frac{M_{cr}}{M_{a}}\right]}^{3}\left[I_{gc}-I_{cr}\right]+I_{cr}$$

Hence, by using the effective moment of inertia $I_{eff}$ obtained from Equation (15), the post-cracking deflection in an RC hollow beam carrying central point loads can be calculated by Equation (16):

(16) $$\delta_{cr}=\frac{1}{48}\frac{PL^{3}}{EI_{eff}}$$

### 6.4. Proposed Equations to Calculate Yield Load and Post-Cracking Deflection

In this section, four different equations are proposed to predicate the yield load ($P_{y-h}$) and the post-cracking deflection ($\delta_{y-h}$) of RC beam with longitudinal opening. The proposed equations are based on the yield load of solid section ($P_{y-s}$), the ratio of net area of hollow section ($A_{t-h}$) to solid section of the beam ($A_{t-s}$), the ratio of compression area of hollow section ($A_{comp-h}$) to compression area of solid section of the beam ($A_{comp-s}$), and, finally, the ratio of uncracked ($I_{ucr}$) to solid transformed moment of inertia of the beam section ($I_{ucr-s}$). Based on the opening dimensions with respect to the beam cross-section dimensions, two equations are proposed for each value of yield load as well as the post-cracking deflection. Equations (17) and (18) are proposed to predicate the yield load value for the hollow section, whereas Equations (19) and (20) are proposed to anticipate the post-cracking deflection.

If bo<b4 or do<d3

(17) $$\frac{P_{y-h}}{P_{y-s}}=0.5\left[\frac{A_{t-h}}{A_{t-s}}+\frac{A_{comp-h}}{A_{comp-s}}\right]$$

If bo≥ b4 or do≥ d3

(18) $$\frac{P_{y-h}}{P_{y-s}}=0.5\left[\frac{A_{t-h}}{A_{t-s}}\frac{I_{ucr}}{I_{ucr-s}}+\frac{A_{comp-h}}{A_{comp-s}}\right]$$

If bo<b4 or do<d3

(19) $$\frac{\delta_{y-h}}{\delta_{y-Eq16}}=\frac{2}{\left[\frac{A_{t-h}}{A_{t-s}}+\frac{A_{comp-h}}{A_{comp-s}}\right]}$$

If bo≥ b4 or do≥ d3

(20) $$\frac{\delta_{y-h}}{\delta_{y-Eq16}}=\frac{4}{{\left[\frac{A_{t-h}}{A_{t-s}}+\frac{A_{comp-h}}{A_{comp-s}}\right]}^{2}\;}$$

To validate the proposed equations, a comparison between the analytical results from FE models and the theoretical values calculated using Equations (17) to (20) is presented in Table 7. The comparison was made for all FE beam models having a longitudinal opening only. Those beams were B1 to B4, B7-1 to B7-6, and B12-1 to B12-3. The minimum and maximum percentage of error found for predicating the yield load were 0.01% and 0.70%, respectively. For anticipating the post-cracking deflections, the minimum and maximum percentage of error found were 0.19% and 2.87%, respectively. From these results, it can be concluded that the equations could calculate the yield load and post-cracking deflection for the concrete beams with longitudinal opening with an acceptable degree of accuracy.

## 7. Conclusions

The response of the RC beams with openings at different positions was investigated. An FE model was built and validated using the experimental results of three RC beams with different LH sizes. The validated FE model was used extensively to establish a parametric study. The parametric study focused on the effect of opening positions, opening dimensions, and beam span on a beam’s response, load capacity, and mode of failure. Accordingly, the following conclusions were drawn:

1. The initial stiffness of the solid beams and beams with openings at different positions was similar. In contrast, the effects of the opening’s dimensions and position on the beam’s load capacities and failure modes were obvious.
2. For simulated beams with TNCH, as the opening height and width increased, the beam loads decreased. The highest value of maximum load reduction was for beam B5-6 with TNCH size = 150 mm × 150 mm (*µ_u_* = 37.21%). All the beams with TNCH failed due to Ys followed by CC except for B5-6 (failed with CC).
3. For simulated beams with TNSH, as the opening width was less than 18.18% of the beam shear span (550 mm), the TNSH opening had trivial effects on the beam’s load capacities (*µ_u_* = 1.26%). All the simulated beams with TNSH failed due to SH except for beam B6-1 (failed with Ys).
4. The simulated beams with LH failed due to Ys followed by CC. The maximum *µ_u_*% for these beams was 17.82% (opening size = 25% of beam size).
5. At *b* = 100 mm, the beams with TNSH experienced the lowest *µ_u_*% for all the studied values of *h*/*H*%. Conversely, the beams with TNCH experienced the highest values of *µ_u_*%. These findings assured the higher effect of the TNCH opening on the flexural capacity than the TNSH. The same previous finding was observed for TNSH and TNCH with *b* = 150 mm. Moreover, the *µ_u_*% increased as *h* increased, regardless of the opening position.
6. The opening position effects on the *µ_u_*% values decreased as the *b* value increased to 150 mm, especially when *h*/*H*% ≥ 1/3. At the same value of *h*, beams with TNCH experienced higher *µ_u_*% values than beams with TNSH. In contrast, increasing *b*/*L_sh_* increased *µ_u_*% for beams with TNSH with higher values than beams with TNCH, especially for *b*/*L_sh_* = 27.27% and *h*/*H*% ≥ 1/3, which assured the high effect of the width of TNSH on the beam shear capacity.
7. The TNSH with 100 mm width had slight effects on the load capacities of solid beams with a 2200 mm span length, similar to that with a short length, although their failure modes were different. The LH decreased the load capacities of beams with a 2200 mm span length compared to the corresponding beams with a 1100 mm span length while the mode of failure was similar.
8. The proposed equations for calculating the yield load and the post-cracking deflection were dependent on the percentages of opening dimensions to beam cross-section dimensions. The proposed equations calculated the yield load and post-cracking deflection for the concrete beams with longitudinal opening to an acceptable degree of accuracy.

## Figures and Tables

**Table 1:** Mechanical properties of the steel materials.

Diameter (mm)	*f_sy_* (MPa)	*f_su_* (MPa)	*E_s_* (GPa)	Elongation%
8	345	411	198	22.1%
10, 12	427	522	209	20.5%

*f_sy_* = steel yield stress, *f_su_* = steel ultimate strength, *E_s_* = steel modulus of elasticity.

**Table 2:** Details of the tested RC beams.

Beam No.	Beam Dimensions, mm		LH Dimensions, mm	
	B	H	*b*_1_	*h*_1_
B1	200	300	0	0
B2	200	300	50	40
B3	200	300	50	80

**Table 3:** Experimental results of the tested RC beams.

Beam No.	*P_cr_*, kN	*P_y_*, kN	*P_u_*, kN	*µ_u_*%	*w_cr_*, mm	Failure Mode
B1	40.6	144.4	160	0	2	Ys
B2	39.2	141.7	155	3.1	3.5	Ys + CC
B3	38.8	137.8	154	3.1	4	Ys + CC

**Table 4:** Comparison between the experimental and FEA results of the tested RC beams.

Beam ID	Experimental Results		FEA Results		Comparisons	
	*P_y,Exp._* (kN)	*P_u,Exp._* (kN)	*P_y,FEA_* (kN)	*P_u,FEA_* (kN)	$\frac{P_{y,Exp.}-P_{y,FEA}}{P_{y,Exp.}}\times 100$	$\frac{P_{u,Exp.}-P_{u,FEA}}{P_{u,Exp.}}\times 100$
B1	144.4	160.9	146.7	166.1	1.6%	3.2%
B2	141.7	155.0	144.3	159.7	1.8%	3.0%
B3	137.8	154.0	141.2	156.7	2.5%	1.1%

**Table 5:** Details of the simulated beams with different openings.

Group No.	Beam ID	*L* (mm)	*b*_1_ (mm)	*h*_1_ (mm)	*b*_2_ (mm)	*h*_2_ (mm)	*b*_3_ (mm)	*h*_3_ (mm)	Test Variable
Control-1	B1	1100	0	0	0	0	0	0	CB
Control-2	B4	2200	0	0	0	0	0	0	Span length (L)
Group 1	B5-1		0	0	100	50	0	0	Transversal near center opening (TNCH)
	B5-2		0	0	100	100	0	0	
	B5-3	1100	0	0	100	150	0	0	
	B5-4		0	0	150	50	0	0	
	B5-5		0	0	150	100	0	0	
	B5-6		0	0	150	150	0	0	
Group 2	B6-1		0	0	0	0	100	50	Transversal near support opening (TNSH)
	B6-2		0	0	0	0	100	100	
	B6-3		0	0	0	0	100	150	
	B6-4	1100	0	0	0	0	150	50	
	B6-5		0	0	0	0	150	100	
	B6-6		0	0	0	0	150	150	
Group 3	B7-1		80	50	0	0	0	0	Longitudinal opening (LH)
	B7-2		80	100	0	0	0	0	
	B7-3		80	150	0	0	0	0	
	B7-4	1100	100	50	0	0	0	0	
	B7-5		100	100	0	0	0	0	
	B7-6		100	150	0	0	0	0	
Group 4	B8-1		80	50	100	50	0	0	LH and TNCH
	B8-2	1100	80	100	100	100	0	0	
	B8-3		80	150	100	150	0	0	
Group 5	B9-1		80	50	0	0	100	50	LH and TNSH
	B9-2	1100	80	100	0	0	100	100	
	B9-3		80	150	0	0	100	150	
Group 6	B10-1	2200	0	0	100	50	0	0	TNCH and L
	B10-2		0	0	100	100	0	0	
	B10-3		0	0	100	150	0	0	
Group 7	B11-1		0	0	0	0	100	50	TNSH and L
	B11-2	2200	0	0	0	0	100	100	
	B11-3		0	0	0	0	100	150	
Group 8	B12-1	2200	100	50	0	0	0	0	LH and L
	B12-2		100	100	0	0	0	0	
	B12-3		100	150	0	0	0	0	
Group 9	B13-1		80	50	100	50	0	0	LH, TNCH, and L
	B13-2	2200	80	100	100	100	0	0	
	B13-3		80	150	100	150	0	0	
	B14-1		80	50	0	0	100	50	
Group 10	B14-2	2200	80	100	0	0	100	100	LH, TNSH, and L
	B14-3		80	150	0	0	100	150	

**Table 6:** The FEA results of the simulated beams with different opening.

Group ID	Beam ID	*P_cr_* (kN)	*P_y_* (kN)	*P_u_* (kN)	*µ_cr_* (%)	*µ_y_* (%)	*µ_u_* (%)	Failure Mode
CB_1_	B1	41.5	144.4	160.9	-	-	-	Ys
CB_2_	B4	36.6	78.1	93.0	-	-	-	Ys + CC
Group 1	B5-1	39.8	134.6	145.2	4.10	8.25	12.58	Ys + CC
	B5-2	25.4	122.4	137.9	38.80	16.56	16.98	Ys + CC
	B5-3	23.9	112.6	119.2	42.41	23.24	28.24	Ys + CC
	B5-4	39.1	133.9	142.3	5.78	8.73	14.33	Ys + CC
	B5-5	24.3	121.9	134.7	41.45	16.91	18.90	Ys + CC
	B5-6	21.5	99.2	104.3	48.19	32.38	37.21	CC
Group 2	B6-1	40.5	149.1	164.1	2.41	0.41	1.20	Ys
	B6-2	39.14	148.8	164.9	5.69	0.61	0.72	SH
	B6-3	36.9	139.9	164	11.08	4.64	1.26	SH
	B6-4	39.3	147.2	164.6	5.30	0.34	0.90	SH
	B6-5	36.8	-	142.6	11.33	-	14.15	SH
	B6-6	32.7	-	115.7	21.20	-	30.34	SH
Group 3	B7-1	38.8	139.2	158.5	6.51	5.11	4.58	Ys + CC
	B7-2	37.6	134.5	153.2	9.40	8.32	7.77	Ys + CC
	B7-3	36.2	130.5	145.2	12.77	11.04	12.58	Ys + CC
	B7-4	38.2	137.5	145.9	7.95	6.27	12.16	Ys + CC
	B7-5	36.0	131.5	140.8	13.25	10.36	15.23	Ys + CC
	B7-6	34.1	125.7	136.5	17.83	14.31	17.82	Ys + CC
Group 4	B8-1	24.5	134.2	146.9	40.96	8.52	11.56	Ys + CC
	B8-2	23.1	123.7	135.6	44.34	15.68	18.36	Ys + CC
	B8-3	20.9	113.3	120.6	49.64	22.77	27.39	Ys + CC
Group 5	B9-1	37.6	142.6	152	9.40	2.79	8.49	Ys + SH
	B9-2	35.1	-	127	15.42	-	23.54	SH
	B9-3	31.5	-	120.8	24.10	-	27.27	SH
Group 6	B10-1	31.0	60.2	78.9	15.3	22.9	15.2	Ys + CC
	B10-2	30.5	64.2	77.1	16.7	17.8	17.1	Ys + CC
	B10-3	30.0	69.1	74.2	18.0	11.5	20.2	Ys + CC
Group 7	B11-1	36.0	78.1	88.9	1.1	0.0	4.4	Ys + CC
	B11-2	36.0	78.1	88.9	1.1	0.0	4.4	Ys + CC
	B11-3	36.0	78.1	88.9	1.1	0.0	4.4	Ys + CC
Group 8	B12-1	32.7	73.0	75.5	10.7	6.5	18.8	Ys + CC
	B12-2	32.0	69.75	72.7	12.6	10.7	21.8	Ys + CC
	B12-3	31.6	66.90	71.1	13.7	14.3	23.5	Ys + CC
Group 9	B13-1	27.4	69.3	74.3	25.1	11.3	20.1	Ys + CC
	B13-2	27.0	63.8	69.4	26.2	18.3	25.4	Ys + CC
	B13-3	26.8	60.4	64.9	26.8	22.7	30.2	Ys + CC
Group 10	B14-1	33.2	73.7	80.6	9.3	5.6	13.3	Ys + CC
	B14-2	32.6	71.1	78.4	10.9	9.0	15.7	Ys + CC
	B14-3	32.1	69.0	77.3	12.3	11.7	16.9	Ys + CC

Ys = steel yielding, CC = concrete crushing, and SH = shear failure.

**Table 7:** Comparison between FE and proposed equation results.

Beam ID	(1)	(2)	(3)	(4)	(5)	(6)
	FE	Theoretical	(%) of Error	FE	Theoretical	(%) of Error
	*P_y_*	*P_y_*		*δ* y	*δ* y	
	kN	kN		mm	mm	
B1	146.7	146.68	0.01%	1.577	1.58	0.19%
B2	144.3	143.598	0.49%	1.604	1.65	2.87%
B3	141.2	141.01	0.13%	1.685	1.71	1.48%
B7-1	139.2	139.04	0.11%	1.48	1.49	0.68%
B7-2	134.5	134.296	0.15%	1.5	1.51	0.67%
B7-3	130.5	129.59	0.70%	1.52	1.53	0.66%
B7-4	137.5	137.08	0.31%	1.54	1.56	1.30%
B7-5	131.5	131.15	0.27%	1.67	1.7	1.80%
B7-6	125.7	125.54	0.13%	1.695	1.71	0.88%
B4	78.1	78.09	0.01%	6.75	6.77	0.30%
B12-1	73	72.98	0.03%	6.95	7.03	1.15%
B12-2	69.75	69.82	0.10%	7.25	7.3	0.69%
B12-3	66.9	66.83	0.10%	7.32	7.38	0.82%

## Data Availability

Not included; the data presented in this article is obtained from an experimental study conducted by authors.

## Nomenclature

$A_{comp-h}$	Compression area of hollow section	*f_su_*	Steel ultimate stress
$A_{comp-s}$	Compression area of beam solid section	*f_sy_*	Steel yield stress
$A_{h}$	Area of opening	*H*	Beam height
$A_{st}$	Area of tension steel	*h_1_*	Opening height of longitudinal opening
$A_{t}$	Area of uncracked transformed section	*h_2_*	Opening height of near the beam center
$A_{t-h}$	Net area of hollow section	*h_3_*	Opening height located near the support
$A_{t-s}$	Solid section of the beam	*I_eff_*	Effective moment of inertia
B	Beam width	*I_g_*	Gross moment of inertia
*b_1_*	Width of longitudinal opening	*I_ucr_*	Moment of inertia of uncracked section
*b_2_*	Width of near the beam center opening	$I_{ucr-s}$	Solid transformed moment of inertia of the beam section
*b_3_*	Width of near the support opening	*L*	Beam length
*c*	Height of the compression zone	$M_{cr}$	Cracking bending moment
c′	Distance between opening center and top concrete	*m*	Modular ratio
*E_s_*	Modulus of elasticity value of steel	*P*	The central applied load
*f_cr_*	Modulus of rupture value of concrete	*P_cr_*	Beam critical load
*f_su_*	Steel ultimate stress	*P_u_*	Beam ultimate load
$\delta_{y-h}$	Post-cracking deflection	*P_y_*	Yield load of solid section
$\delta_{pcr}$	Central deflection at pre-cracking stage		
